# Model Fitting Versus Curve Fitting: A Model of Renormalization Provides a Better Account of Age Aftereffects Than a Model of Local Repulsion

**DOI:** 10.1177/2041669515613669

**Published:** 2015-11-18

**Authors:** Sean F. O’Neil, Amy Mac, Gillian Rhodes, Michael A. Webster

**Affiliations:** Department of Psychology, University of Nevada, Reno, Nevada, USA; ARC Centre of Excellence in Cognition and its Disorders, School of Psychology, University of Western Australia, Perth, Australia; Department of Psychology, University of Nevada, Reno, Nevada, USA

**Keywords:** Face perception, age perception, adaptation, aftereffects, norm-based coding

## Abstract

Recently, we proposed that the aftereffects of adapting to facial age are consistent with a renormalization of the perceived age (e.g., so that after adapting to a younger or older age, all ages appear slightly older or younger, respectively). This conclusion has been challenged by arguing that the aftereffects can also be accounted for by an alternative model based on repulsion (in which facial ages above or below the adapting age are biased away from the adaptor). However, we show here that this challenge was based on allowing the fitted functions to take on values which are implausible and incompatible across the different adapting conditions. When the fits are constrained or interpreted in terms of standard assumptions about normalization and repulsion, then the two analyses both agree in pointing to a pattern of renormalization in age aftereffects.

## Introduction

Aftereffects of adaptation to faces have been widely studied, in part to try to examine the ways in which the visual system represents information about the face. Two widely debated alternatives to this representation are a norm-based code, in which individual faces are represented relative to how they deviate from an average or prototype, which is therefore special; and an exemplar or multichannel code, in which each face is encoded by mechanisms tuned to the absolute characteristics defining the face, with no face special (Valentine, Lewis, & Hills, 2015). These alternatives predict very different patterns of aftereffects (Rhodes et al., 2005; Webster & MacLeod, 2011). Yet distinguishing between them is difficult, partly because these patterns depend on sampling the aftereffects at multiple levels along a face dimension. Recently, we took advantage of the fact that facial age is among the few facial dimensions that observers can readily scale (by assigning a perceived age) and used this to assess how adaptation to one age affects the appearance of all others (O’Neil, Mac, Rhodes, & Webster, 2014). We found that the aftereffects for young and old faces were characterized by approximately uniform shifts, while middle-aged faces judged as a norm for age produced little aftereffect. We concluded from this pattern that adaptation to facial age is consistent with a simple norm-based code. Storrs (2015) challenges this conclusion, arguing that the aftereffects we reported are equally or better described by a pattern of *local repulsion* predicted by a multichannel model. Here, we show that her analyses were based on allowing the curve fits for repulsion to take on unrealistic and inconsistent values for the underlying mechanisms. When the curve fits are constrained by conventional assumptions about the channel models, then normalization provides a better account of the age aftereffects, supporting our original claims.

**Figure 1.**
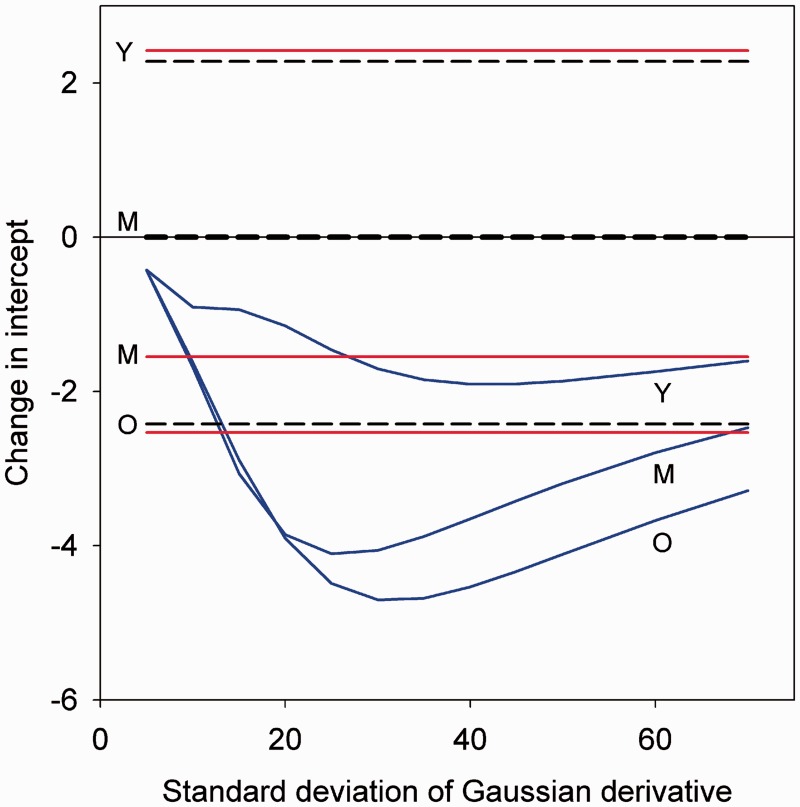
Blue lines: Intercepts of linear regression lines fit to GD curves with different standard deviations. All GDs were adjusted for a peak aftereffect of±2.4, and spanned an age range from 18 to 89, with zero crossings at the young (Y), Middle (M), or old (O) adapting ages. Black lines: intercept shifts for a uniform renormalization of +2.4 (Y), 0 (M), or −2.4 (O). Red lines: measured intercepts for the three adapting conditions.

To understand this debate, consider how the pattern of aftereffects differs for normalization (norm-based coding) and repulsion (multichannel) as commonly construed (e.g., Pond et al., 2013; Storrs & Arnold, 2015; Zhao, Series, Hancock, & Bednar, 2011). The differences were illustrated in Figure 1 of O’Neil et al. (2014). For normalization, the hallmark characteristics are:

- Aftereffects of similar size and in the same direction for all faces including the adapting face, consistent with a shift in the appearance of the adapting face toward the norm. Thus when adapting to a young face, all faces should look older.
- No aftereffect when adapting to the norm.
- Asymmetries in the aftereffects, such that adapting to an older or younger face alters the norm, while adapting to the norm does not bias the perceived age of old and young faces.

For repulsion, the defining pattern is:

- Changes in opposite directions for faces on opposite sides of the adapting level. Faces younger than the adapt age should appear even younger, and faces older than the adapt age should appear even older.
- No change in the perceived age of the adapting level itself.
- A similar pattern at all adapting levels, and thus no asymmetries in the aftereffects between different ages.

In our original report, we evaluated these alternatives by comparing regression lines fit to the age estimates before or after adaptation. Normalization predicts approximately constant shifts in the judgments, changing the intercept but not the slope of the function, while adapting to the norm should produce neither a slope nor intercept change. Moreover, normalization predicts the intercept changes should be in opposite directions for young and old adaptors. This is the pattern we tested for and found. The perceptual expansion produced by repulsion instead predicts a steepening of the slope and predicts that intercepts would always be shifted and in the same direction (toward more negative values) for all three adapt levels, a pattern we did not find (see Figure 1). The size of these linear shifts obviously depends on the magnitude and bandwidth of the adaptation. Yet the aftereffects for repulsion never predict the pattern we observed of opposite shifts in the intercepts after adaptation to young versus old faces. From this we again conclude that the observed aftereffects are consistent with the pattern predicted by normalization while inconsistent with the pattern predicted by repulsion and that our original analyses based on testing the slopes and intercepts were sufficient to distinguish between the alternative models.

In her commentary, Storrs raises several critiques of our work. One is that we did not evaluate the aftereffects directly (i.e., the changes in the age ratings before and after adaptation) but instead focused on the absolute ages under the different conditions. However, this misrepresents what we did. While our regression fits were based on the absolute perceived ages, the analyses we conducted explicitly assessed whether the slopes or intercepts of the regression lines changed with adaptation. This is necessarily an assessment of the aftereffect (of the change between pre- and postadapt settings) and one which is equivalent to asking whether the aftereffect itself was constant across age (i.e., a change in the intercept without a change in slope, the basic effect we found).

**Figure 2.**
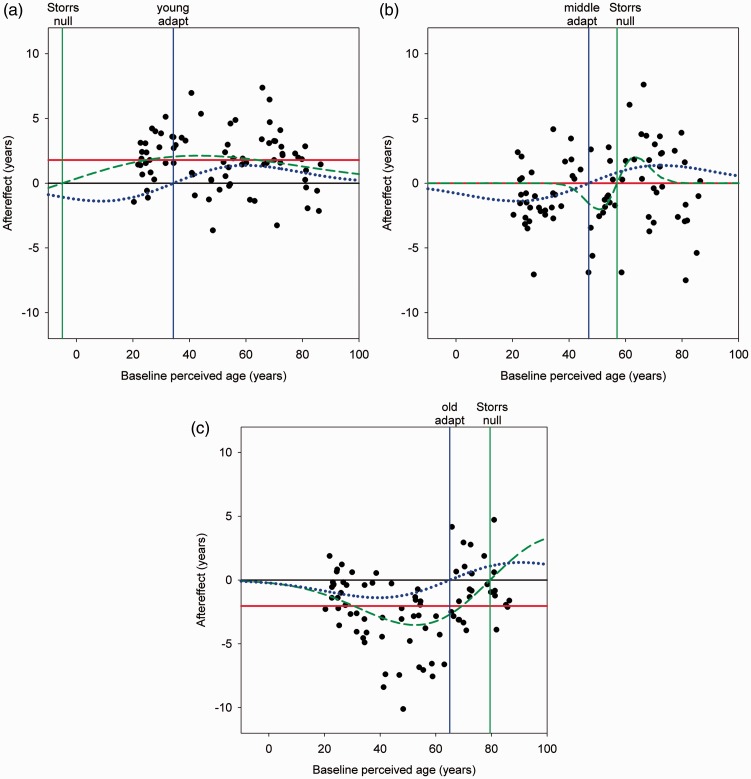
Model and curve fits for the three adapting conditions. Symbols show the aftereffects (post vs. pre settings for each face) as a function of the preadapt perceived age. Vertical blue lines show the mean age of the adapting faces. Green dashed lines: best fitting GD curve, reproduced from Storrs. Vertical green lines show the age at the zero-crossings for the independent GD curve fits (Storrs null). Blue dotted line: best fitting standard repulsion model. Red solid line: best fitting standard normalization model.

A second critique was that our analysis did not provide a fair test of a repulsion model. To pursue this, Storrs instead compared how well the aftereffects were fit by a linear function versus a Gaussian derivative (GD). Her choice of the GD was reasonable for simulating repulsion but allowing the function to take on any values is not. In particular, Storrs separately fit each adapting condition with a function that was free to vary in its amplitude, bandwidth, and zero-crossing. To see why this is a problem, consider first the fits she obtained to the young adapting age (Figure 2(a), green dashed line). Here, observers were adapted to faces in their 30’s, but the best fit occurs when the repulsion is centered not on the adapt age, but instead crosses zero at an ‘*age*’ of *negative* 5 years (4 decades from the adapt level), and also has a very broad standard deviation (48 years). What should we conclude from this? First, we argue that Storrs’ analysis is not testing a viable *model* of adaptation. If it were, then the conclusion would be that when adapting to the age of young adults, the sensitivity losses were largest in channels tuned to an impossibly younger age—to how old we looked before our parents met. Her analysis is instead an exercise in *curve* fitting, in which the parameter values are not constrained to have a mechanistic interpretation. Second, Storrs’ argument is that this curve provides as good an estimate of the aftereffects as a linear fit. However, this is not because very different functions fit the data as well, but because the two fitted functions she estimated are in fact very similar over the finite span of adult ages (18–89) at which the aftereffects were actually measured. As Figure 2(a) shows, a GD function with very broad tuning and a very remote zero-crossing approximates a linear change over the range of interest. In this regard it is not surprising that an arbitrary GD can fit the data at least as well, because it includes an approximation to locally linear effects as a possible solution. In fact this is the reason her repulsion fit predicts the positive intercept change we reported for the young adapt condition—because over the range of physically possible ages the repulsion is all in the same direction. Finally, the fact that this roughly linear effect best describes the aftereffects also for the GD curve shows that Storrs’ own analysis yields the essential pattern of aftereffects predicted by normalization. Specifically, both the GD and linear curve fits predict that adapting to faces in their 30’s biases all adult ages—including the adapting age—to appear older.

A similar problem occurs for the older adapting age (Figure 2(c)). Here again the best-fitting GD function does not have a zero crossing at the adapt level (65), but is shifted now to older ages (79; a difference as large as the age differences between our adapting conditions), and the function again predicts aftereffects broadly similar to the pattern predicted by normalization (i.e., after adapting to older faces, almost all faces—including the adapt faces—appear younger). In this case, the tuning is narrower (*SD* = 27) and the fits are not as well captured by a constant change at all ages, a point noted in our original paper. Yet the still very broad and consistent direction of the predicted aftereffects is again closer to the hallmark pattern of normalization than repulsion.

The middle-age adapt condition reveals a further flaw in Storrs’ analysis, which arises because she fit each of the three adapting conditions independently (Figure 2(b)). Here again the zero-crossing for the best-fitting GD curve is displaced from the expected null at the adapting level (47 vs. 57). Yet more importantly, in this case, the bandwidth of the function is now much narrower (6.5). This leads to fits for the GD functions which are internally inconsistent as a model of the aftereffects. While the bandwidths of channels could plausibly vary at different stimulus levels, they cannot simultaneously be so broad that young and old adapt ages induce strong aftereffects in middle-ages (and each other), yet so narrow that middle-age adaptation does not induce complementary repulsion on the young and old ages. However, as curve fits, this asymmetry between the aftereffects for middle ages versus young and old ages once more follows the pattern of aftereffects expected by normalization. As her fitted GD functions predict, adapting to the young or old faces biased middle-ages, while adapting to this norm did not produce corresponding biases in the appearance of the young or old ages. Thus while Storrs argues that her analysis contradicts our conclusions, simple inspection of the fitted functions shows that she has instead confirmed that the aftereffects for all three adapt conditions follow the standard pattern predicted for normalization, and not for repulsion.

What if we instead compare viable models of normalization versus repulsion, by constraining the fitted functions to embody the characteristics that define conventional implementations of these models? For repulsion, we used the GD function but forced the null to coincide with the adapting level to reflect the hallmark of typical repulsion aftereffects. Further, we required the strength and spread of the adaptation to be the same at each adapting level, to preclude the implausibly large asymmetries in Storrs’ independent curve fits. This left two free parameters controlling the magnitude and bandwidth of the GD function. For normalization, we assumed a very simple model in which there is no adaptation to the norm, while adapting to younger or older ages produces a constant bias at all test ages. Consistent with evidence from distortion aftereffects (Susilo, McKone, & Edwards, 2010), we further required that the strength of the aftereffect increase in proportion to how far the adapt age was from the norm. This leaves only a single free parameter controlling the magnitude of the aftereffect. Fits from the two models are plotted in Figure 2 as the red (normalization) and blue (repulsion) curves. Despite fewer degrees of freedom, the model of normalization provides significantly better fits to the aftereffects than the model of repulsion (RMS error = 2.66 for normalization and 2.90 for repulsion, vs. 3.08 for no model). This was assessed with a Wilcoxon-signed rank test of the relative errors for the two models, which makes minimal assumptions and does not penalize the repulsion model for having more free parameters (across all three adapt conditions combined: *z* = 3.06, *p* = .002). The normalization model also outperformed the repulsion model for both the younger (RMS error = 2.14 for normalization and 2.63 for repulsion, vs. 2.79 for no model; *z* = 3.42, *p* < .001) and older (RMS error = 2.78 for normalization and 3.09 for repulsion, vs. 3.45 for no model; *z* = 2.14, *p* = .033) adapt conditions, while the two models did not differ from each other for the middle-age adapt (RMS error = 2.96 for normalization and 2.91 for repulsion; *z* = 0.187, *p* = .853), for which normalization equals the prediction for no aftereffects. Thus, this analysis again confirms that the aftereffects are better described by normalization than repulsion, at least when based on standard and viable forms of these aftereffects.

The adaptation effects we measured were weak and it remains possible that elaborations of either model might better describe the actual pattern of age aftereffects (e.g., Elliott, Georgeson, & Webster, 2011). In fact, the normalization and repulsion models differ only quantitatively and seamlessly in properties such as the number and bandwidth of the mechanisms (Webster & MacLeod, 2011). However, the point we emphasize is that any model comparisons should be based on realizable neural models, and not on curve fits in which the parameters are allowed to take on implausible or impossible values for the underlying adapted mechanisms. When the aftereffects are fit or interpreted in this way, then both our analyses and Storrs’ own are better captured by normalization.
